# Severe Cutaneous Adverse Reaction to Lamotrigine: A Case of Stevens-Johnson Syndrome in a Psychiatric Patient

**DOI:** 10.7759/cureus.78812

**Published:** 2025-02-10

**Authors:** Kajomi Shingala, Dipesh Nariya

**Affiliations:** 1 Department of Dermatology, Shri M P Shah Government Medical College, Jamnagar, IND; 2 Department of Pharmacology, Shri M P Shah Government Medical College, Jamnagar, IND

**Keywords:** antiepileptic drugs (aed), cutaneous adverse drug reaction, dermatological emergencies, medication safety, stevens-johnson syndrome (sjs)

## Abstract

Stevens-Johnson syndrome (SJS) is a severe and potentially life-threatening mucocutaneous reaction often triggered by medications. Antiepileptic drugs, particularly lamotrigine, are recognized as significant causative agents. Early identification and management are crucial to improve patient outcomes.

We report the case of a 26-year-old male diagnosed with schizoaffective bipolar disorder who developed SJS following the dose escalation of lamotrigine. He presented with multiple well-defined erythematous lesions, targetoid macular lesions, oral erosions, and fever. Based on clinical findings and a detailed medication history, lamotrigine was identified as the probable causative agent using the World Health Organization (WHO)-Uppsala Monitoring Centre (UMC) causality assessment scale. Laboratory investigations revealed elevated inflammatory markers and Severity-of-Illness Score for Toxic Epidermal Necrolysis (SCORTEN) assessment predicted a significant mortality risk. Management included immediate discontinuation of lamotrigine, systemic corticosteroids, antihistamines, antibiotics, and topical agents for symptomatic relief. Supportive care led to gradual re-epithelialization, resolution of mucosal lesions, and eventual discharge with residual post-inflammatory hyperpigmentation.

This case emphasizes the risk of severe cutaneous adverse reactions with lamotrigine, particularly within the initial weeks of treatment. The pathophysiology of SJS involves immune-mediated keratinocyte apoptosis, with granulysin playing a key role. Current treatment strategies remain debated, with corticosteroids, cyclosporine, and tumor necrosis factor α (TNF-α) inhibitors showing potential benefits. Early drug discontinuation, vigilant monitoring, and multidisciplinary management are crucial in reducing morbidity and mortality.

This report underscores the need for heightened vigilance when prescribing lamotrigine, particularly during dose escalation. Strengthening pharmacovigilance, patient education, and screening for genetic predispositions may help mitigate the risk of drug-induced SJS. Further research into optimal therapeutic strategies is warranted to improve clinical outcomes in affected patients.

## Introduction

Stevens-Johnson syndrome (SJS) is a rare, acute, and potentially life-threatening mucocutaneous disorder characterized by widespread epidermal necrosis and detachment. It arises from a delayed hypersensitivity reaction, typically triggered by medications such as sulfonamides, antiepileptics, allopurinol, and nonsteroidal anti-inflammatory drugs (NSAIDs). Other causes include infections (e.g., Mycoplasma pneumoniae, herpesviruses, human immunodeficiency virus [HIV]) and noninfectious conditions such as systemic lupus erythematosus and collagen vascular diseases.

SJS and toxic epidermal necrolysis (TEN) are now considered part of a disease continuum, with severity classified based on total body surface area (TBSA) involvement: SJS (<10% TBSA), SJS-TEN overlap (10-30% TBSA), and TEN (>30% TBSA). Involvement of two or more mucosal surfaces is a crucial diagnostic feature, distinguishing SJS from other dermatoses.

The condition carries a significant risk of mortality and long-term complications. The mortality rate is approximately 5% for SJS and up to 30% for TEN, with an increased risk persisting even one year after the acute episode. Survivors often experience long-term sequelae, including ocular damage, chronic skin changes, pulmonary complications, and renal or hepatic impairment [1]. Genetic predisposition also plays a role, with certain human leukocyte antigen (HLA) alleles linked to drug-induced SJS/TEN in specific ethnic populations [2]. Early recognition and aggressive supportive management are critical to improving outcomes. The Severity-of-Illness Score for Toxic Epidermal Necrolysis (SCORTEN) and Age, Bicarbonate, Cancer, Dialysis, 10% Body Surface Area (ABCD-10) scores serve as essential prognostic tools, guiding risk assessment and therapeutic decisions.

## Case presentation

**Figure 1:**
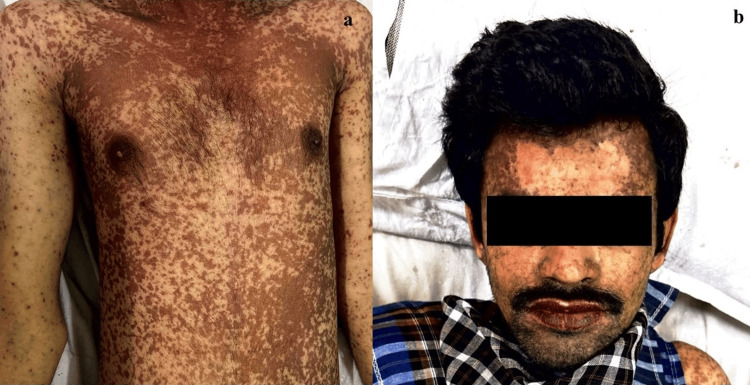
Dermatological examination at initial presentation a: Erythematous lesions involving upper extremities and torso. b: Erythematous lesions over the face and crusted lesions over the lips

A 26-year-old male patient reported to the Dermatology outpatient department with multiple well-defined erythematous lesions all over the body. The patient had multiple well-defined erythematous targetoid macules and patches, bilaterally symmetrical, ranging from 0.2 × 0.4 cm to 0.5 × 2 cm, distributed over the face, upper limbs, and torso. Erosive lesions with an erythematous base (0.15 × 1 cm to 2 × 3 cm) were noted on the back. The lips were inflamed with multiple crusted lesions. Oral mucosal involvement included well-defined to ill-defined erythematous lesions with white plaques, causing mild pain (Figure 1). The patient complained of fever for the past four days. There was no previous history of drug-induced reactions or any dermatological diseases. The patient was admitted to the dermatology ward for further management. Vitals measured on admission were temperature 102 F, pulse rate 128 beats/min and blood pressure 122/84 mmHg. Systemic examination revealed no further abnormalities. Pain as assessed using universal pain assessment tool was found to be zero (no pain). Based on a detailed medication history, presenting symptoms, and clinical examination, a provisional diagnosis of drug-induced SJS was made during the initial outpatient evaluation.

Several dermatological conditions were considered in the differential diagnosis. Erythema multiforme (EM) presents with target lesions but lacks significant epidermal detachment. Generalized bullous fixed drug eruption (GBFDE) shows localized recurrent lesions upon re-exposure. Staphylococcal scalded skin syndrome (SSSS), common in children, lacks mucosal involvement and shows subcorneal cleavage on biopsy. Autoimmune blistering disorders (e.g., pemphigus vulgaris) have a chronic course and distinct histopathology. Toxic shock syndrome (TSS) can mimic SJS but is caused by bacterial exotoxins and presents with severe hypotension. Based on the acute onset, widespread targetoid lesions, mucosal involvement, and positive drug history, drug-induced SJS was confirmed.

As per the previous history of the patient, he presented to the psychiatry outpatient department with chief complaints of irritability, aggressive behavior, anger outbursts, and self-muttering behavior. Upon detailed mental status examination, the provisional diagnosis of schizoaffective bipolar disorder was established. The patient was admitted to the psychiatry ward in the tertiary care teaching hospital. Upon admission, oral treatment was started with lamotrigine 25 mg once daily (OD), sertraline 25 mg OD, and risperidone 1 mg twice daily (BD). The patient was maintained on the same treatment for three days. Daily psychiatric evaluation and mental status examination were carried out to assess the response to the treatment. The dose of lamotrigine was increased to 25 mg BD on day four of the treatment to manage the symptoms of bipolar disorder. The patient was discharged from the psychiatry ward three days later with a total duration of hospital admission of seven days.

The patient was on treatment with lamotrigine, sertraline, and risperidone with the above-mentioned doses at home. After two weeks of starting treatment with lamotrigine, the patient started developing symptoms. Lamotrigine was suspected to be the causative agent for this adverse drug reaction (ADR). Causality assessment of ADR was done using the World Health Organization (WHO)-Uppsala Monitoring Centre (UMC) causality assessment scale and there was a ‘probable’ association between the suspected medication and ADR in our case. Lamotrigine was immediately withdrawn.

Management of the patient was started with admission under a strict thermoregulatory and monitoring environment, and a series of hematological and biological investigations were carried out. Complete blood count, serum electrolytes, hepatic and renal panel, and basic metabolic panel were normal. Urine routine and microscopic examinations were normal. Erythrocyte sedimentation rate (ESR) and C-reactive protein (CRP) were elevated (40 mm at first hour and 48 mg/l respectively). Investigations for hepatitis B surface antigen (HBsAg) and hepatitis C virus (HCV) were negative. HIV I and II (antigen and antibody) tests were non-reactive. Prognosis was assessed by employing the SCORTEN scoring system. Based on age 26 years, detached body surface area >10 %, serum bicarbonate 21 mEq/L, serum urea nitrogen 21 mg/dl, serum glucose (random blood sugar [RBS]) 87mg/dl, heart rate 128/minute and no signs of malignancy, SCORTEN was calculated to be 3 (mortality rate of 35.8%). The elevated ESR and CRP are consistent with the inflammatory nature of the syndrome (Table 1).

**Table 1:** Laboratory Findings HBsAg: hepatitis B surface antigen, HCV: hepatitis C virus, RBS: random blood sugar

Laboratory Findings	Findings in this case	Normal Range
Erythrocyte Sedimentation Rate	40 mm/hour	0-15 mm/hour
C-reactive Protein	48 mg/dl	0-1 mg/dl
HBsAg	Negative	-
HCV Antibody	Negative	-
HIV 1 & 2 Antigen and Antibody	Negative	-
Serum bicarbonate	21 mEq/L	22-29 mEq/L
Serum Urea Nitrogen	21 mg/dl	6-24 mg/dl
Serum glucose (RBS)	87 mg/dl	<125 mg/dl

Therapeutic management included supportive measures with IV dexamethasone (8 mg, 2 cc), IV pheniramine (45.5 mg, 2 cc), and IV ceftriaxone (2 g once daily), along with IV fluid replacement including electrolyte correction, and pain management. The same injectable treatment was continued for seven days. Oral treatment with capsule Becosules (vitamin B complex, vitamin C) and capsule omeprazole (20 mg) was prescribed twice a day. Tablet nimesulide (100 mg) and tablet cetirizine (10 mg) were prescribed twice a day. 

Topical treatment was prescribed based on lesion type and location. For skin lesions, gentian violet lotion, betamethasone cream, and framycetin cream were advised twice daily. For oral mucosal involvement, clotrimazole and liquid glycerin were applied three times daily. Gargles with lidocaine viscous solution and chlorhexidine solution three times a day were advised and continued for one week.

**Figure 2:**
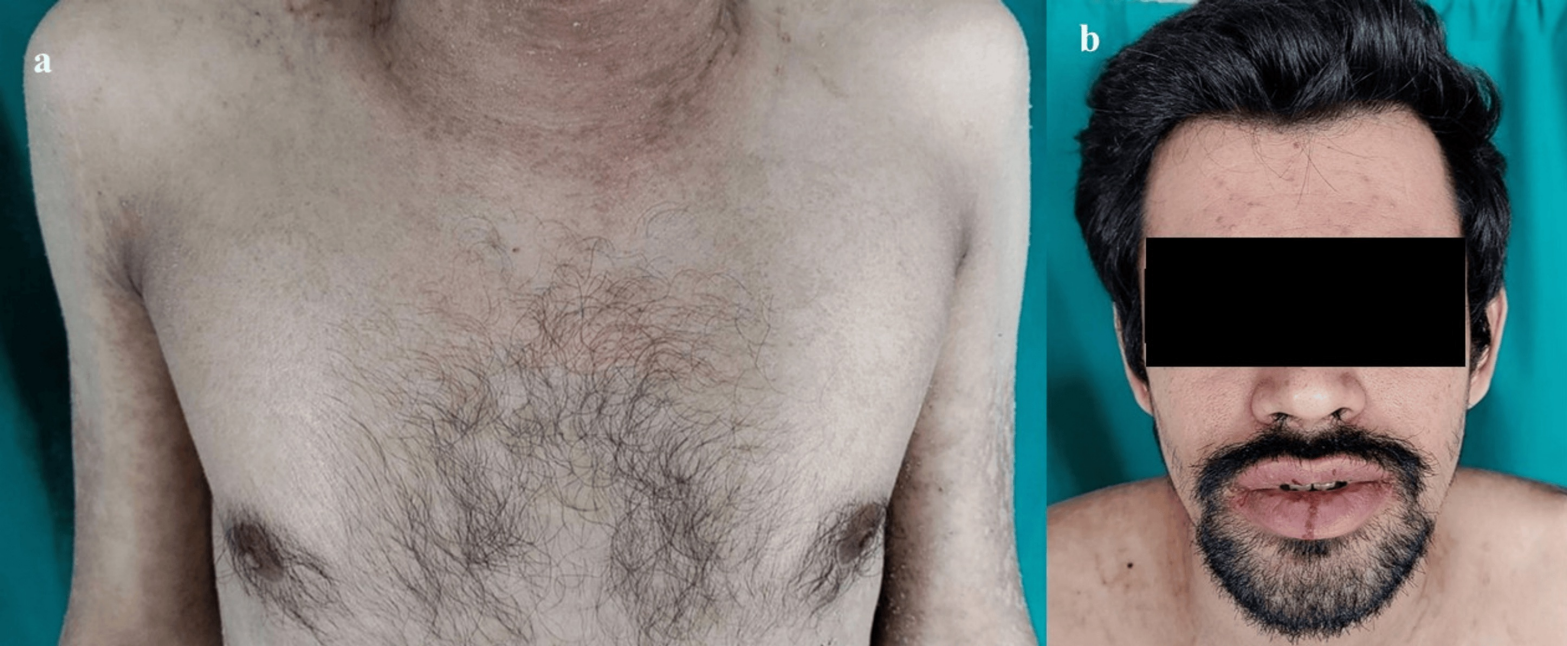
Dermatological examination on the day of discharge a: Resolution of the lesions and re-epithelialization of skin over torso, b: Resolution of lesions over the face and lips

Detailed daily assessment and close monitoring of the patient was carried out to guide further treatment. Lesions of oral mucosa started resolving and gradually improved compared to initial presentation. Temperature was normalized, skin re-epithelialized gradually and mucosal lesions regressed over the course of treatment. Residual post-inflammatory hyperpigmentation was noted over some areas (Figure 2); they were managed with medications on discharge. The patient was discharged on the 10th day of admission with significant clinical improvement.

## Discussion

Certain antiepileptic drugs (AEDs) have additional FDA-approved uses beyond epilepsy, such as treating neuropathic pain, migraines, anxiety, insomnia, and bipolar disorder [3]. Consequently, these medications are widely prescribed for various conditions. However, AEDs, particularly lamotrigine and carbamazepine, are linked to severe skin reactions like SJS and TEN [4,5]. Notably, these two drugs carry black box warnings due to the risk of such reactions.

Lamotrigine is indicated for the maintenance treatment of bipolar I disorder in adults (18 years and older). It helps delay the recurrence of mood episodes, including depression, mania, hypomania, and mixed episodes, in patients who have been stabilized with standard acute-phase therapy. When considering any increase in the dose of lamotrigine, the escalation regimen for Lamictal in adults with bipolar disorder should be followed. As suggested in the Lamictal (lamotrigine) US FDA label, the risk of severe, potentially life-threatening rash may be increased by coadministration of lamotrigine with valproate, exceeding the recommended initial dose of lamotrigine, or exceeding the recommended dose escalation. Any dose escalation of lamotrigine needs close monitoring to detect early manifestations of SJS [4].

Six antiepileptic drugs had the highest estimated risk of SJS and toxic epidermal necrolysis: lamotrigine, carbamazepine, phenytoin, zonisamide, rufinamide, and clorazepate, according to an analysis of reports in the FDA Adverse Event Reporting System [6]. The findings underscore the need for neurologists to warn patients of the potential risks for severe skin complications with these drugs [1]. Greater than 90% of SJS/TEN reactions associated with AEDs occur within the first two months of treatment initiation [7].

The pathophysiology of SJS is not fully understood, but it is characterized by widespread keratinocyte apoptosis. This process is believed to involve several mediators in a cell-mediated cytotoxic reaction, with T lymphocytes and natural killer (NK) cells likely playing a central role in triggering apoptosis [8]. Other mediators, such as Fas-Fas ligand, tumor necrosis factor α (TNF-α), and perforin-granzyme B, have also been implicated. However, recent research suggests that granulysin may be the primary mediator responsible for keratinocyte destruction. Studies have identified granulysin as the most abundantly expressed cytotoxic molecule in the blisters of SJS patients, with its levels correlating directly with the severity of the disease [9].

The SCORTEN scale is used to predict mortality in SJS-TEN cases. It evaluates factors such as patient age, presence of malignancy, tachycardia, elevated urea levels, serum glucose, bicarbonate levels, and the percentage of total body surface area affected. Additionally, the ABCD-10 score is another prognostic tool that assesses age, bicarbonate levels, cancer history, dialysis requirement, and percentage of epidermal detachment, providing further risk stratification in SJS-TEN cases. A SCORTEN score exceeding 4 indicates a 90% mortality risk [10]. SJS is a life-threatening condition with a global mortality rate ranging from 10% to 34%, emphasizing the need for prompt recognition and treatment through a multidisciplinary approach [11].

The management in our case included immediate discontinuation of the suspected offending agent and treatment with injectable corticosteroids, antibiotics, and antihistamines to rapidly attenuate the acute inflammation. Topical agents employed in our case included antiseptics and corticosteroids. Prevention of secondary bacterial infection is of paramount importance due to the high mortality associated with infected cases of SJS. Despite the high SCORTEN score, early intervention and aggressive supportive care led to a successful recovery, with the patient being discharged in stable condition. This case highlights the importance of timely management in improving outcomes, even when the predicted mortality risk is high.

Identifying and stopping the suspected medication is critical to halt disease progression. Medical therapies for SJS/TEN include corticosteroids, cyclosporine, intravenous immunoglobulin (IVIG), and TNF-α inhibitors. The use of corticosteroids in managing SJS/TEN remains a topic of debate. A meta-analysis of immunomodulatory therapies, encompassing 96 studies, highlighted the potential benefits of corticosteroids in improving prognosis. The analysis also found cyclosporine to show promising results, whereas other treatments, such as IVIG, did not demonstrate significant benefits [12]. A randomized controlled trial involving 91 patients compared TNF-α inhibitor (etanercept) with corticosteroids for SJS-TEN management. Mortality rates in both groups were lower than those predicted by the SCORTEN scale (8.3% for the etanercept group and 16.3% for the corticosteroid group, compared to 17.7% predicted by SCORTEN) [13]. Further research is recommended to explore the role of etanercept in managing SJS/TEN.

Laboratory tests, including liver and kidney function panels, play a crucial role in assessing systemic involvement and guiding management in SJS. While the patient's results were normal, these markers are essential for monitoring drug metabolism, detecting early organ dysfunction, and assessing disease severity, as SJS can lead to hepatic and renal complications due to systemic inflammation, dehydration, and sepsis risk. Regular monitoring helps in preventing complications and optimizing supportive care.

Strengthening monitoring systems to detect and prevent adverse drug reactions could reduce the incidence of SJS, saving costs in the long run. For high-risk drugs like carbamazepine, lamotrigine and allopurinol, genetic screening (e.g. HLA-B*1502 testing) could prevent SJS, though the upfront costs might limit its feasibility in countries with limited resources like India. Ensuring the availability of affordable generics for supportive care can help reduce treatment costs. Data on the economic burden of SJS in India and other low- and middle-income countries (LMICs) is limited. Conducting pharmacoeconomic studies is essential to guide resource allocation and policymaking for better management strategies.

## Conclusions

This case highlights the importance of early recognition and management of lamotrigine-induced SJS, a potentially life-threatening condition. Prompt withdrawal of the suspected drug, combined with supportive care and a multidisciplinary approach, resulted in a favorable outcome for the patient. The case underscores the need for heightened vigilance during dose escalation of medications like lamotrigine and emphasizes the role of close monitoring to prevent severe adverse drug reactions. Further awareness and preventive strategies, including pharmacovigilance and patient education, are essential to mitigate the risks associated with drug-induced SJS.
